# Correlation of serum apolipoprotein B with the Framingham Risk Score among a group of Iraqi subjects: a cross-sectional study

**DOI:** 10.7717/peerj.19883

**Published:** 2025-08-18

**Authors:** Israa Nather Ahmed, Fatimatuzzahra’ Abd Aziz, Raid Dhia Hashim

**Affiliations:** 1 Discipline of Clinical Pharmacy, School of Pharmaceutical Sciences, Universiti Sains Malaysia, Penang, Malaysia; 2 Department of Pharmacy, Al-Rasheed University College, Baghdad, Iraq; 3 Department of Clinical Chemistry, College of Pharmacy, Al-Farahidi University, Baghdad, Iraq

**Keywords:** Apolipoprotein B, Framingham Risk Score, Cutoff point, Coronary artery disease risk

## Abstract

**Background:**

Apolipoprotein B (apoB) is the primary structural protein in low-density lipoprotein (LDL) and plays a crucial role in atherogenesis. The Framingham Risk Score (FRS) is a widely used tool for assessing cardiovascular disease (CVD) risk. However, the correlation between apoB and FRS in Iraqi individuals remains underexplored.

**Objectives:**

This study aims to evaluate the association between serum apoB levels and FRS, establishing its potential utility as a predictive biomarker for coronary artery disease (CAD) risk.

**Methods:**

A cross-sectional study was conducted on 201 individuals aged ≥30 years attending a clinical laboratory in Baghdad between November 2022 and October 2023. Serum apoB and lipid profiles were measured, and FRS was calculated for all participants. Correlation analysis between apoB and FRS was performed using Spearman’s test, while group comparisons were conducted *via* Kruskal–Wallis and Mann–Whitney tests. The predictive performance of apoB for high FRS was assessed using receiver operating characteristic (ROC) analysis, determining an optimal cutoff value.

**Results:**

The median age of participants was 48 years, with males constituting 51.2% of the cohort. Median apoB and FRS values were 130 mg/dL and 4, respectively. A strong positive correlation was observed between serum apoB and FRS (*R* = 0.8, *P* = 0.0001). ROC analysis identified a cutoff value of 97.75 mg/dL for apoB in predicting high CAD risk.

**Conclusions:**

These findings suggest that apoB may serve as a reliable biomarker for CAD risk assessment in the Iraqi population, where its predictive value has been underexplored. The identified cutoff value (97.75 mg/dL) highlights its potential role in refining risk stratification beyond traditional lipid markers. Further prospective studies are needed to validate these findings and assess their clinical impact.

## Introduction

Apolipoprotein B (apoB) is a key structural component of all atherogenic lipoproteins, including low-density lipoprotein (LDL), playing a pivotal role in lipoprotein assembly, receptor binding, and metabolic regulation. As the primary structural protein of LDL, apoB is fundamental to hepatic lipoprotein synthesis and is directly implicated in atherogenesis (Olofsson, Wiklund & Borén, 2007).

Cardiovascular disease (CVD) remains a leading cause of morbidity and mortality (Gaidai, Cao & Loginov, 2023), with diabetes mellitus, hypertension, and dyslipidemia being the primary risk factors for coronary artery disease (CAD) (Duggan et al., 2022). Addressing these risk factors is central to clinical practice and preventive cardiology (Wong et al., 2022). Several algorithms have been developed to estimate CAD risk, including the Reynolds Risk Score (Ridker et al., 2007), Pooled Cohort Equations (Lloyd-Jones et al., 2019), and the Framingham Risk Score (FRS). The FRS, derived from the landmark Framingham Heart Study, is a widely adopted tool for estimating the 10-year risk of CAD in middle-aged, asymptomatic individuals (Andersson et al., 2019; Dehghan et al., 2024). It incorporates key risk variables such as age, sex, total cholesterol (TC), high-density lipoprotein cholesterol (HDL-C), systolic blood pressure, antihypertensive therapy, and smoking status (D’Agostino Sr et al., 2008). Despite the widespread use of the FRS, its reliance on conventional lipid parameters raises concerns. Previous studies have demonstrated a significant proportion of CAD patients exhibit normal lipid profiles (Silverman et al., 2016; Hoog et al., 2022), requesting the predictive accuracy of standard risk equations. Notably, serum apoB has demonstrated a stronger association with CAD development than traditional lipid measures (Lawler et al., 2017). Unlike low density lipoprotein cholesterol (LDL-C), which estimates cholesterol content, apoB directly quantifies the number of atherogenic lipoprotein particles, offering a more precise assessment of atherogenic burden (Sniderman, Pedersen & Kjekshus, 1997). From a pathophysiological standpoint, cholesterol within non-HDL particles is only harmful when transported by apoB-containing lipoproteins (Skålén et al., 2002). Studies found that each lipoprotein particle that penetrates the endothelium of the coronary artery can initiate atherosclerosis regardless of its cholesterol content (Tabas, Williams & Borén, 2007), and as the number of atherosclerotic lipoprotein particles increases there will be an increase in the retention of lipoprotein particles in the arterial wall where the latter can induce inflammation and leads to an increase in the coronary artery permeability and makes the endothelium layer loss its barrier function (Khalil, Wagner & Goldberg, 2004). The best example for this explanation is the stronger correlation of atherogenicity with the small dense LDL particles compared to normal-size LDL particles although small-dense LDL particles carry less amount of cholesterol in comparison to normal-size LDL particles (Froyen, 2021). Additionally, apoB measurement is cost-effective, does not require fasting, and remains reliable even in the presence of hypertriglyceridemia or reduced LDL-C levels due to lipid lowering therapy (Glavinovic et al., 2022). Approximately 20% of individuals with metabolic disorders, including hypertriglyceridemia, type 2 diabetes, and obesity, exhibit discordance between apoB and LDL-C levels, underscoring the limitations of LDL-C-based risk assessment (Glavinovic et al., 2022). Unlike LDL-C and non-HDL-C, which are derived through mathematical estimation, apoB is directly measured in laboratories, offering greater precision.

In recent years, increasing attention has been given to the pathophysiological role of serum apoB in CAD progression, particularly in explaining the presence of CAD in individuals with an accepted range of lipid profiles (Mach et al., 2020). However, despite its well-established role in atherogenesis, most previous studies have examined apoB in the context of diagnosed CAD, rather than as a predictor of future risk. To our knowledge, only one study has directly correlated apoB with the FRS (Ryoo et al., 2011). Given these insights, our study explores the correlation between serum apoB levels and FRS in an Iraqi cohort, aiming to clarify its utility in CAD risk prediction and primary prevention. This approach offers an added advantage by assessing apoB’s role in risk stratification before the clinical onset of CAD, especially in a population that remains underrepresented in cardiovascular research.

## Methods

### Study design and setting

This cross-sectional study was conducted at a general private clinical laboratory (Dr. Abas Abd Almuaed Laboratory) in Baghdad between November 2022 and October 2023. The study adhered to the Strengthening the Reporting of Observational Studies in Epidemiology (STROBE) (Von Elm et al., 2007) guidelines to ensure methodological rigor and transparency.

### Subject recruitment and eligibility criteria

Participants were recruited consecutively from individuals attending the laboratory for routine check-ups. The study included adults aged ≥30 years who were fasting for 8–12 h before blood collection and who voluntarily agreed to participate. Exclusion criteria included individuals with a history of CVD, except for hypertension, and those with chronic illnesses such as diabetes mellitus or chronic kidney disease. Individuals with acute illness, those currently receiving medical treatment (except for hypertension), pregnant or breastfeeding women, and those who declined to share personal data were also excluded.

### Sample size determination and participant selection

**Figure 1:**
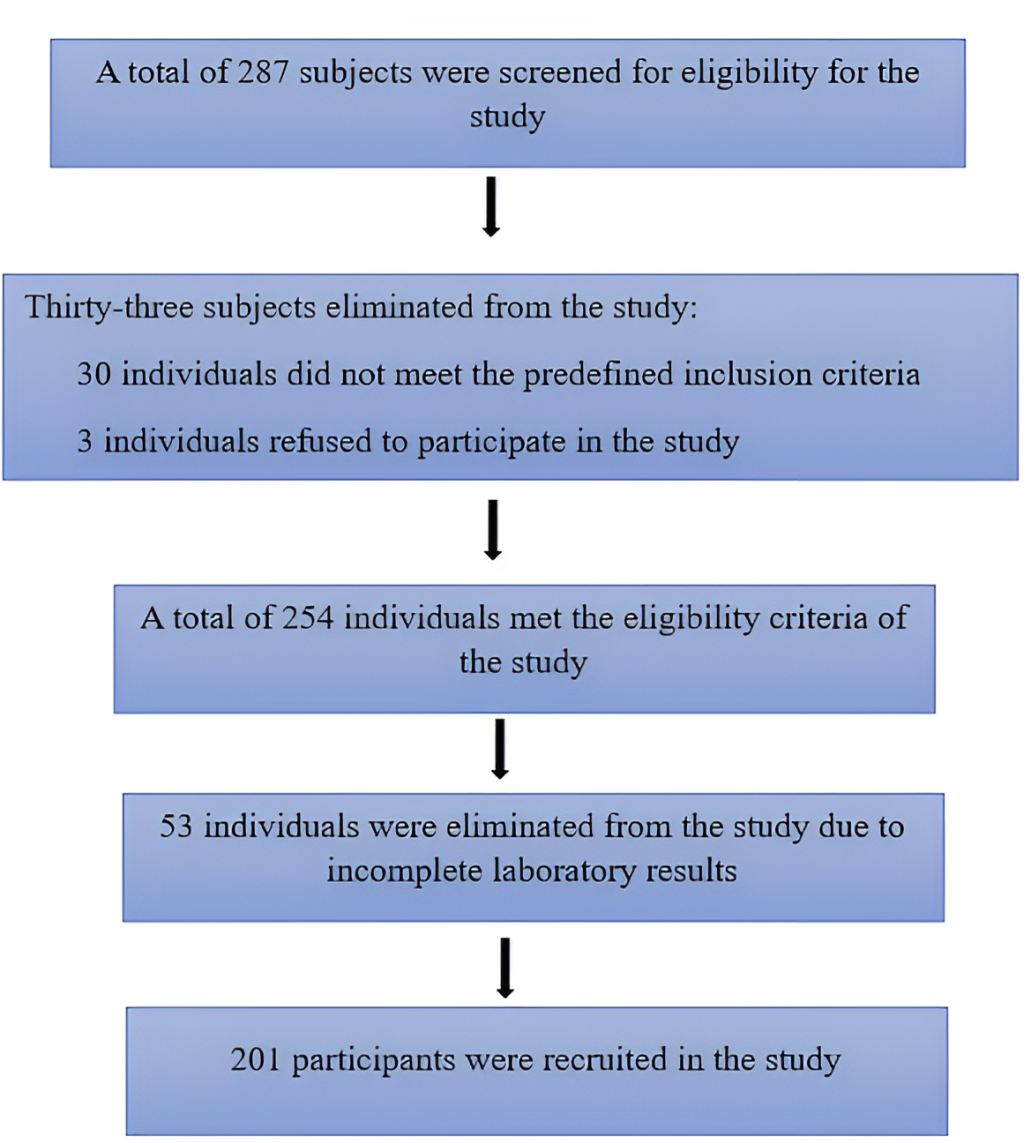
Flowchart of participant recruitment process.

The required minimum sample size was estimated using a modified two-mean formula with a power of 80% and a Confidence Interval of 95%. The mean and the standard deviation of serum apoB in the three FRS groups were derived from a previous study (Ryoo et al., 2011). The equation determined that at least 120 subjects were necessary to achieve adequate statistical power. A total of 287 participants were initially screened. After applying the eligibility criteria, 86 individuals were excluded, comprising 15 with diabetes, two pregnant women, 13 with a history of CVD, and three who declined participation. An additional 53 participants were excluded due to incomplete data, resulting in a final study population of 201 eligible participants. These exclusions were due to technical issues such as hemolyzed, clotted, or insufficient blood samples, which rendered biochemical analysis incomplete. Since the exclusions were based solely on laboratory sample quality and not participant characteristics, they are unlikely to have introduced selection bias. Figure 1 illustrates the participant selection process, detailing the exclusions due to eligibility criteria, refusal, and incomplete data.

### Data collection and clinical measurements

Demographic and clinical data were collected from all participants. Age, sex, and smoking status were recorded as key cardiovascular risk factors. Blood pressure (BP) was measured using the Korotkov method while participants were in a seated position, using a calibrated aneroid sphygmomanometer. The mean of two consecutive BP readings was used for analysis. Weight and height were measured using a digital scale and stadiometer, respectively, and body mass index (BMI) was calculated.

### Framingham risk score calculation

The 10-year risk of CAD was estimated for each participant using the FRS equation. This calculation was based on seven key cardiovascular risk factors, including age, sex, TC, HDL-C, systolic blood pressure, use of antihypertensive therapy, and smoking status. Based on the calculated FRS, participants were categorized into three risk groups: those with a low risk (≤10%), those at moderate risk (10–20%), and those classified as high risk (>20%). The FRS was determined using a validated computerized Excel program (https://qxmd.com/calculate/calculator_253/framingham-risk-score-atp-iii), ensuring accuracy and standardization in risk assessment.

### Laboratory analysis

All participants underwent fasting blood sample collection *via* venipuncture of the antecubital vein. Blood samples were immediately processed within the same laboratory to ensure standardization. TC, TG, and HDL-C were measured using an enzymatic colorimetric method on a Roche C311 autoanalyzer. VLDL-C was calculated as TG/5, while non-HDL-C was determined by subtracting HDL-C from TC. LDL-C was estimated using the Friedewald equation (Tremblay et al., 2004). Serum apoB levels were assessed using an enzyme-linked immunosorbent assay (ELISA) (BT LAB Human Apolipoprotein B, apo-B ELISA Kit, ab 190806) and they were stored at −20 °C for a maximum of one month to be analyzed collectively. The excess blood specimen was treated daily by an autoclave and collected in special containers by a representative of the Ministry of Health of Iraq, where the tainted samples were disposed of according to the Iraqi laboratory guidelines. All instruments were validated by daily quality control and calibration as needed; tools were validated by calibration as required.

### Statistical analysis

Data were analyzed using SPSS version 26 (IBM Corp., Armonk, NY, USA). The Kolmogorov–Smirnov test was performed to assess normality, revealing a non-normal distribution of the data. Therefore, continuous variables were presented as medians with interquartile ranges (IQRs), while categorical variables were expressed as numbers and percentages. To compare continuous variables between groups, Kruskal–Wallis and Mann–Whitney *U* tests were used. To account for multiple comparisons in subgroup analyses, a correction for multiple testing was applied using the Bonferroni method where appropriate.

Spearman’s correlation test was employed to determine the association between apoB levels and FRS. To evaluate the predictive performance of apoB for high CAD risk (≥20% FRS), receiver operating characteristic (ROC) curve analysis was conducted and the optimal cutoff value for apoB was determined based on sensitivity and specificity. The area under the curve (AUC) was calculated to assess the discriminative ability of apoB, A *P*-value <0.05 was considered statistically significant for all analyses.

### Ethical considerations

This study was approved by the Research and Ethics Committee of Universiti Sains Malaysia (protocol code: USM/JEPeM/22060348) and the AL-Rasheed University College Ethics Committee (approval number: RUCPD30122201). All participants provided written informed consent before enrollment, and data confidentiality was maintained throughout the study.

## Results

### Descriptive analysis

The baseline characteristics of the 201 participants are summarized in Table 1. The median age of the study population was 48 years, with males representing 103 (51%) of the participants. The median serum apoB level and FRS score were 130 mg/dL and 4, respectively. Based on FRS classification, participants were categorized into low-FRS (131, 65.2%), moderate-FRS (37, 18.4%), and high-FRS (33, 16.4%) groups.

**Table 1:** Baseline characteristics of participants.

	Total (***N*** = **201**)	Male (*N* = 103)	Female (*N* = 98)	*P*-value
Variable	**Median (IQ range)**	**Median (IQ range)**	**Median (IQ range)**	
Age (year)	48 (34–63)	51 (37–63)	41 (33–54)	**0.001**
BMI (kg/m^2^)	31.6 (28–34)	31 (27–34)	32 (28–35)	0.1
Systolic Bp (mmHg)	124 (110–140)	125 (110–140)	124 (110–139)	0.9
Diastolic Bp (mmHg)	66 (60–90)	79 (60–90)	74.5 (60–89)	0.07
TC (mg/dl)	199 (170–220)	200 (180–221)	190 (165–220)	0.1
TG (mg/dl)	158 (107–212)	189 (125–227)	138 (95–190)	**0.0001**
LDL-C (mg/dl)	120 (96–140)	125 (100–141)	118 (92–138)	0.1
VLDL-C (mg/dl)	30.8 (21–42)	37 (24–45)	27 (19–37)	**0.0001**
HDL-C (mg/dl)	43 (36–51)	41 (35–49)	45 (37–53)	**0.006**
Non-HDL-C (mg/dl)	153 (125–178)	159 (130–183)	147 (116–172)	**0.03**
apoB (mg/dl)	130 (82–184)	148 (126–260)	87 (72–136)	**0.0001**
FRS	**4 (1–15)**	**13 (6–21)**	**1 (1–4)**	**0.0001**
	**Total (***N*** = **201**)**	**Male**	**Female**	*****P***-value**
Variable	***N*(%)**	***N*(%)**	***N*(%)**	
Hypertension	84 (41.8%)	53 (51.5%)	31 (31.6%)	**0.004**
Smoking	100 (49.8%)	64 (62.1%)	36 (36.7%)	**0.0001**
FRS Risk Groups				
<10% (Low Risk)	131 (65.2%)	41 (39.8%)	90 (91.8%)	**0.0001**
10–20% (Moderate Risk)	37 (18.4%)	34 (33%)	3 (3.1%)	**0.0001**
>20% (High Risk)	33 (16.4%)	28 (27.2%)	5 (5.1%)	**0.0001**

**Notes.**

N, number of participants.

*P*-values of <0.05 are set in bold and present significant differences.

BMI body mass index TC total cholesterol TG triglyceride LDL-C low-density lipoprotein cholesterol VLDL-C very low-density lipoprotein cholesterol HDL-C high-density lipoprotein cholesterol non-HDL-C non-high-density lipoprotein cholesterol apoB apolipoprotein B FRS Framingham Risk Score FRS groups: ≤10% low risk 10%–20% moderate risk ≥20% high risk

Mann–Whitney *U* test compared the medians of age, BMI, systolic Bp, diastolic Bp, TC, LDL-C, TG, VLDL-C, HDL-C, non-HDL-C, apoB, and FRS between males and females, Chi-square test compared the number and percentages of hypertension, smoking and FRS groups between males and females.

A comparison of measured variables between males and females revealed that males had significantly higher median values for age (51 *vs.* 41 years, *p* < 0.05), apoB (148 *vs.* 87 mg/dL, *p* < 0.05), and FRS (13 *vs.* 1, *p* < 0.05). Moreover, males exhibited significantly higher levels of triglycerides (189 *vs.* 138 mg/dL, *p* < 0.05), VLDL-C (37 *vs.* 27 mg/dL, *p* < 0.05), and non-HDL-C (159 *vs.* 147 mg/dL, *p* < 0.05). The prevalence of hypertension (51.5% *vs.* 31.6%, *p* < 0.05) and smoking (62.1% *vs.* 36.7%, *p* < 0.05) was also greater among males. Regarding cardiovascular risk stratification, a higher proportion of males fell into the intermediate (FRS 10–20%; 33% *vs.* 3.1%, *p* < 0.05) and high-risk (FRS > 20%; 27.2% *vs.* 5.1%, *p* < 0.05) categories compared to females.

### Clinical characteristics associated with FRS groups

The measured variables were compared across the three FRS groups as shown in Table 2. Significant differences were observed except for BMI, which did not vary significantly. Age, TC, LDL-C, TG, and VLDL-C were significantly higher in participants with higher FRS scores, while HDL-C was significantly lower in those with higher FRS.

**Table 2:** The measured variables across 10-years predicted risk groups.

	FRS ≤ 10% (1) *N* = 131	FRS 10–20% (2) *N* = 37	FRS ≥ 20% (3) *N* = 33	(1&2) *P*-value	(2&3) *P*-value	(1&3) *P*-value	(1, 2, &3) *P*-value
Age (year)	38 (32–48)	63 (54–66)	67 (56–72)	**0.0001**	0.08	**0.0001**	**0.0001**
BMI (kg/m2)	31 (28–35)	30 (26–34)	31 (29–33)				0.5
TC (mg/dl)	189 (166–217)	197 (170–210)	212 (198–240)	0.8	0.002	**0.0001**	**0.001**
TG (mg/dl)	141 (97–198)	167 (106–211)	207 (188–225)	0.2	0.003	**0.0001**	**0.0001**
HDL-C (mg/dl)	45 (38–53)	42 (34–50)	33 (30–37)	0.2	**0.0001**	**0.0001**	**0.0001**
LDL-C (mg/dl)	113 (91–134)	121 (82–140)	138 (125–161)	0.4	0.003	**0.0001**	**0.0001**
VLDL-C (mg/dl)	28 (19–39)	31 (21–41)	41 (37–45)	0.4	0.004	**0.0001**	**0.0001**
Non-HDL-C (mg/dl)	144 (119–167)	151 (113–170)	183 (164–211)	0.7	**0.0001**	**0.0001**	**0.0001**
Apo B (mg/dl)	94 (73–132)	150 (139–167)	310 (283–338)	**0.0001**	**0.0001**	**0.0001**	**0.0001**

**Notes.**

N number of participants BMI body mass index TC total cholesterol TG triglyceride LDL-C low-density lipoprotein cholesterol VLDL-C very low- density lipoprotein cholesterol HDL-C high-density lipoprotein cholesterol non-HDL-C non-high-density lipoprotein cholesterol apoB apolipoprotein B FRS Framingham Risk Score FRS groups: ≤10% low risk 10%–20% moderate risk ≥20% high risk

Kruskal–Wallis Test was used to compare the median values of variables among the three FRS groups (age, BMI, TC, TG, HDL-C, LDL-C, VLDL-C, non-HDL-C, apoB). Mann–Whitney U Test was used to compare the medians between each of the two FRS groups.

The adjusted *p*-value after Bonferroni correction was 0.00139. Significant *p*-values are set in bold.

### Correlation analysis

The correlation between FRS and key metabolic parameters is detailed in Table 3. A strong positive correlation was observed between FRS and both apoB (*r* = 0.8, *p* < 0.05) and age (*r* = 0.7, *p* < 0.05). Additionally, weak to moderate positive correlations were noted between FRS and TC (*r* = 0.35), TG (*r* = 0.4), non-HDL-C (*r* = 0.4), VLDL-C (*r* = 0.38), and LDL-C (*r* = 0.39), all statistically significant. Conversely, a significant negative correlation was observed between FRS and HDL-C (*r* =  − 0.3, *p* < 0.05).

**Table 3:** Correlations of FRS with other variables.

Variables	FRS	
	***r***	***P*-value**
Age	0.7	**0.0001**
BMI	−0.01	0.8
TC (mg/dl)	0.35	**0.0001**
TG (mg/dl)	0.4	**0.0001**
HDL-C (mg/dl)	−0.3	**0.0001**
Non-HDL-C (mg/dl)	0.4	**0.0001**
LDL-C (mg/dl)	0.39	**0.0001**
VLDL-C (mg/dl)	0.38	**0.0001**
apoB (mg/dl)	0.8	**0.0001**

**Notes.**

*N* = 201, r: Spearman correlation coefficient, *P*-value <0.05 was set in bold and presents a significant correlation.

BMI body mass index TC total cholesterol TG triglyceride HDL-C high-density lipoprotein cholesterol non-HDL-C non-high-density lipoprotein cholesterol LDL-C low-density lipoprotein cholesterol VLDL-C very low-density lipoprotein cholesterol apoB apolipoprotein B

Spearman rank-order test was used to analyze correlation.

### Sex- and age-stratified analysis

Sex-based comparisons across clinical and biochemical variables are summarized in Table 1, where males showed significantly higher median values for apoB, total triglycerides, and FRS scores, while females had higher HDL-C levels. To further explore age-related differences in cardiovascular risk markers among females (*n* = 98), we conducted a subgroup analysis stratified by age. As shown in Table 4, apoB levels showed no significant correlation with age among females aged <50 years (*r* = 0.1, *p* = 0.3), whereas a strong positive correlation was observed in females aged ≥50 years (*r* = 0.6, *p* = 0.0001). FRS scores also correlated with age in both groups, with a moderate association in younger females (*r* = 0.4, *p* = 0.001) and a stronger correlation in older females (*r* = 0.7, *p* = 0.0001).

**Table 4:** Correlation of age with serum apoB level and FRS among age groups of females.

Age	*N* = 98	*r*	*p*-value
	Apo B		
Females (<50 years)	67	0.1	0.3
Females (≥50 years)	31	0.6	0.0001
	**FRS**		
Females (<50 years)	67	0.4	0.001
Females (≥50 years)	31	0.7	0.0001

**Notes.**

*N* = 98, r, Spearman correlation coefficient, *P*-value <0.05 was set in bold and presents a significant correlation.

Apo B Apolipoprotein B FRS Framingham risk score

Spearman Rank-Order Test was used to analyze correlation.

### Predictive performance of apoB for high CAD risk

**Figure 2:**
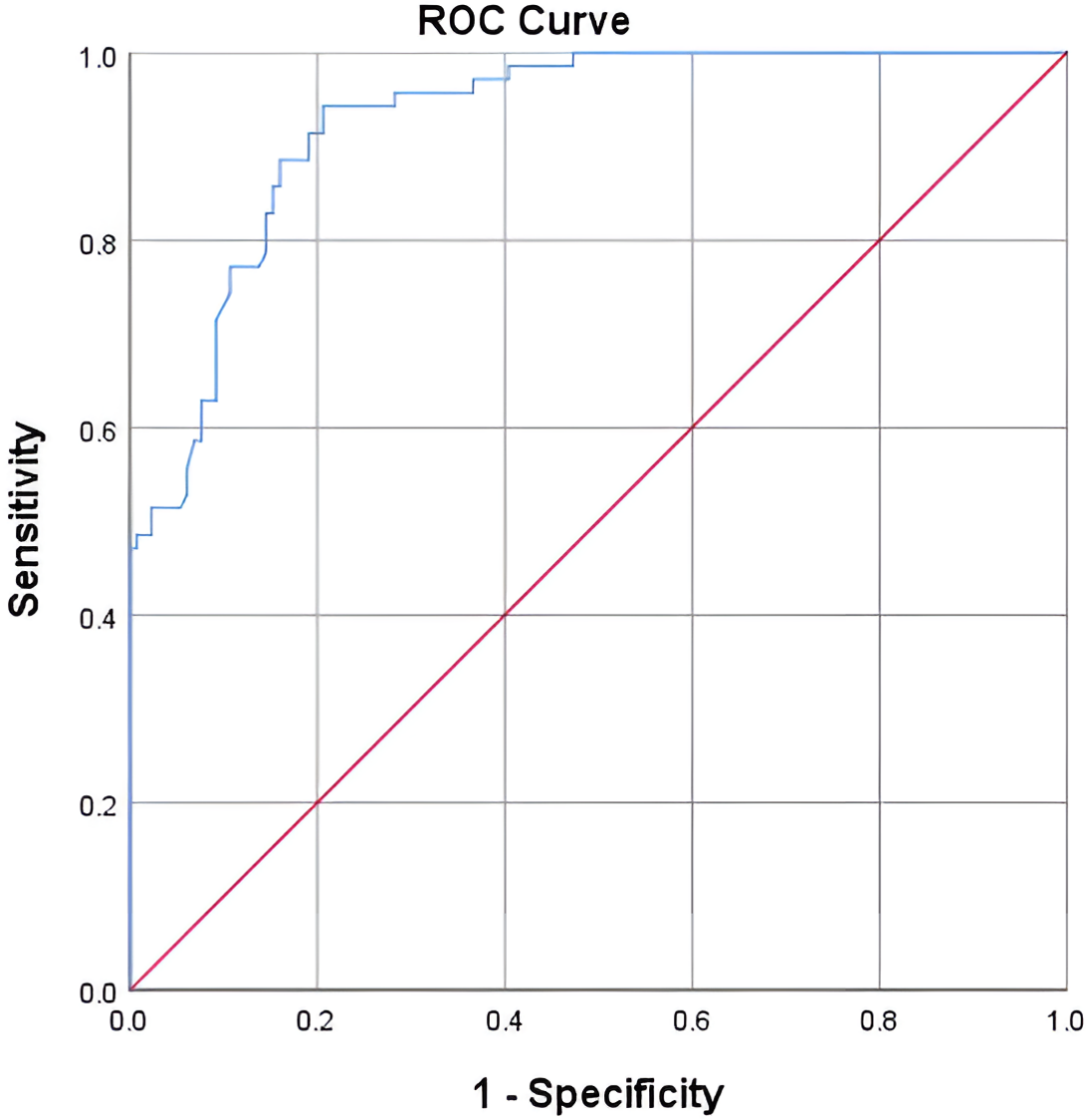
ROC curve for apoB as a prediction tool for 10-year CAD risk as estimated by FRS.

The ROC curve analysis demonstrated that a serum apoB level of 97.75 mg/dL serves as a potential cutoff value for predicting high CAD risk as estimated by FRS as shown in Fig. 2. The AUC was 92% (*p* = 0.0001), indicating strong discriminatory power for CAD risk stratification. Where FRS was classified into two groups FRS <  20% and FRS > 20% where the second group was considered as CAD risk equivalent. apoB value of 97.75 mg/dL was the cutoff point with the highest sensitivity (94%) and specificity (79%) for predicting high FRS (FRS > 20%).

## Discussion

The present study provides compelling evidence that serum apoB levels vary significantly across FRS-defined risk groups, with the highest levels observed in the high-FRS group, followed by the moderate and low-risk groups. This trend, coupled with the strong correlation between apoB and FRS, as well as ROC analysis, suggests a potential role for apoB in refining CAD risk stratification as estimated by FRS. Although the FRS includes total cholesterol and HDL-C, it might not account for the full spectrum of atherogenic lipoproteins. ApoB, by quantifying the number of atherogenic particles directly, offers additional predictive value beyond traditional lipid parameters included in FRS.

Despite the fact that the apoB/apoA1 ratio has also been proposed as a meaningful marker, prior evidence suggests that apoB alone is a superior predictor of CAD mortality, with the predictive power of the ratio largely driven by apoB itself. Therefore, the current study focused on apoB as a stand-alone marker, which is both pathophysiologically grounded (Galimberti, Casula & Olmastroni, 2023; Sierra-Johnson et al., 2009).

The current findings align with a large observational study of 13,523 Korean males, which demonstrated a significant association between serum apoB and CAD risk in individuals without diabetes or hypertension (Ryoo et al., 2011). However, beyond this study, there remains a paucity of research directly correlating apoB with FRS. Instead, most prior investigations have focused on apoB levels in established CAD cases. For instance, a case-control study examining patients with unstable angina diagnosed *via* electrocardiography found a significant association between elevated serum apoB and unstable angina, compared to controls (Al-Tu’ma, Mohammedad & Al-Sarraf, 2017). Similarly, another study involving 160 participants (80 with acute coronary syndrome and 80 controls) confirmed a strong correlation between apoB, apoA, and apoB/A ratio with acute coronary syndrome incidence (Jassim & Ali, 2016). While these studies highlight the role of apoB in diagnosed CAD cases, our study extends this evidence by demonstrating its association with CAD risk prediction *via* FRS. Further supporting our findings, a prospective study of 9,231 asymptomatic adults in Denmark, followed over eight years, reported that apoB outperformed LDL-C in predicting incident CVD (Benn et al., 2007). Likewise, the Health Professionals Follow-up Study, which followed 18,225 men for six years, found that apoB was superior to non-HDL-C in predicting nonfatal myocardial infarction (Pischon et al., 2005). These longitudinal studies reinforce the clinical relevance of apoB in cardiovascular risk assessment, underscoring its potential role as a valuable biomarker in primary prevention. However, apoB is still not recommended by the current guidelines for clinical practice in the primary prevention of CAD.

A key contribution of this study is the identification of a cutoff value of 97.75 mg/dL for predicting high CAD risk using FRS. However, the cutoff value was previously investigated in other populations but the results were controversial. The variation in the cutoff value may be attributed to differences in the design of each study, where the present study has used FRS as a determinant factor of the predictive role of apoB while the previous studies have used the presence or the prospective occurrence of CAD instead. In addition to the ethnicity of participants and the methodology employed. For instance, a case-control study conducted among 437 participants aged 40–70 years old admitted to the hospital for angiography indicated a higher cutoff value of 120 mg/dl for serum apoB for diagnosis of CAD (Levinson, 2007). Another case-control study of 271 participants conducted in Iran reported a lower cutoff value of serum apoB of 89.5 mg/dl for angiography-diagnosed CAD patients (Mashayekhi et al., 2014). Notably, the present cutoff value falls within the range recommended by the American Diabetes Association (ADA) and the American College of Cardiology Foundation (ACCF) for high-risk patients (<90 mg/dL) (Brunzell et al., 2008) and the Adult Treatment Panel III (ATP III) recommendations for moderate-risk individuals (<110 mg/dL) (Grundy, 2002). This positioning suggests that apoB may serve as a clinically meaningful biomarker for risk stratification, bridging the gap between high-risk thresholds established by ADA/ACC and moderate-risk thresholds outlined in ATP III, as both of them have two or more risk factors with no previous history of diabetes or CAD. Despite these similarities, current clinical guidelines from the American Heart Association (AHA) and American College of Cardiology (ACC) do not yet incorporate apoB as a routine parameter for cardiovascular risk assessment. Instead, LDL-C and non-HDL-C remain the primary targets for lipid-lowering therapy. However, the Canadian Cardiovascular Society and the European Society of Cardiology/European Atherosclerosis Society (ESC/EAS) do recommend apoB as a co-primary or secondary target, particularly in patients with elevated triglycerides or those at high risk (Arnett et al., 2019; Pearson et al., 2021; Pirillo, Casula & Catapano, 2023).

Given the strong correlation between apoB and FRS observed in this study, further prospective research is needed to validate apoB’s predictive utility and refine its cutoff values for different risk groups, particularly in Middle Eastern populations.

Sex-based differences in serum apoB levels were evident in this study, with males exhibiting a significantly higher apoB concentration than females. This discrepancy may be attributed to the higher catabolic rate of apoB-containing lipoproteins in females (Matthan et al., 2008), a phenomenon previously observed in a large Swedish cohort of 147,576 participants (Jungner et al., 1998). Furthermore, a strong positive correlation between apoB levels and age was noted, likely due to the age-related decline in LDL receptor activity and reduced catabolism of apoB-containing lipoproteins (Ericsson et al., 1991). This trend aligns with prior research conducted in 100 males and 100 females (Avogaro et al., 1979), reinforcing the notion that aging contributes to increased apoB concentrations. Given that age is a key determinant in FRS calculations, this age-related rise in apoB may partially explain its correlation with FRS in the current study. A more detailed age-stratified analysis among females revealed significantly higher apoB levels in women aged ≥50 years compared to those <50 years. This could be attributed to postmenopausal hormonal changes, particularly the decline in estrogen levels, which has been shown to correlate with higher apoB concentrations (Ej, 1994). Supporting this, previous study had demonstrated a decrease in serum apoB levels following estrogen replacement therapy (Granfone et al., 1992), highlighting the hormonal regulation of apoB metabolism in females.

### Limitations

The current study solely examined the correlation between baseline serum apoB levels and the FRS using a cross-sectional design. As such, no follow-up period was included, and the actual incidence of CAD was not measured. Therefore, while apoB showed a strong association with estimated 10-year CAD risk, it remains uncertain whether changes in apoB levels would influence the future occurrence of CAD. Prospective longitudinal studies are needed to validate these findings with actual clinical outcomes.

## Conclusion

The findings of this study underscore the potential role of serum apoB as a promising predictive biomarker for CAD risk stratification in the Iraqi population, demonstrating a strong positive correlation with FRS. apoB may offer advantages over LDL-C and non-HDL-C in assessing cardiovascular risk, particularly in populations where conventional lipid markers may be less predictive. Unlike previous studies that primarily associated apoB with established CAD, this study contributes population-specific insights by identifying a cutoff value of 97.75 mg/dL for high CAD risk. However, the study’s reliance on FRS as a surrogate measure presents a limitation, as FRS was developed for Western populations and may not fully capture Iraqi-specific risk factors. Further prospective studies are needed to validate these findings and assess apoB’s role in clinical guidelines for CAD risk prediction, dyslipidemia diagnosis, and preventive strategies.

##  Supplemental Information

10.7717/peerj.19883/supp-1 Supplemental Information 1 Dataset

10.7717/peerj.19883/supp-2 Supplemental Information 2 STROBE checklist

## Additional Information and Declarations

### Data Availability

The following information was supplied regarding data availability: Data is available at Zenodo: Nathir Ahmed, I. (2025). Serum Apolipoprotein B [Data set]. Zenodo. https://doi.org/10.5281/zenodo.16412998. Raw data is available in the Supplemental Files.
